# Rare and everywhere: Perspectives on scale-free networks

**DOI:** 10.1038/s41467-019-09038-8

**Published:** 2019-03-04

**Authors:** Petter Holme

**Affiliations:** Institute of Innovative Research, Tokyo Institute of Technology, Nagatsuta-cho 4259, Midori-ku, Yokohama, Kanagawa 226-8503 Japan

## Abstract

Are scale-free networks rare or universal? Important or not? We present the recent research about degree distributions of networks. This is a controversial topic, but, we argue, with some adjustments of the terminology, it does not have to be.

This year marks the 20th anniversary of one of the most influential, but also most controversial, network papers—“Emergence of scaling in random networks” by Barabási and Albert^1^. Together with Watts and Strogatz’ work on small-world networks^2^, it helped connecting network scientists of different backgrounds into an interdisciplinary field^3^, and drew much attention and many aspiring young scientists (including myself) to the study of networks.

Barabási and Albert found that for three empirical networks “independent of the system and the identity of its constituents, the probability *P*(*k*) that a vertex in the network interacts with *k* other vertices decays as a power law”^1^. They attributed this to a growth mechanism, preferential attachment, where new vertices attach to old ones with a probability proportional to the degree (number of neighbors) of the old vertices. Within just a few years, a multitude of papers claimed to have discovered new types of networks with power-law degree distributions—scale-free networks—and new mechanisms creating them^4^.

In the early days, the appeal of scale-free networks came from complexity science^3^—an umbrella term for many research directions trying to find hidden laws in the complex world around us, and simple rules to explain them. One such idea is emergence—the phenomenon that a multitude of interacting units can as a group behave in ways not predictable from the behavior of the units alone. Examples of emergence include the undulating movements of a school of fish or a bird flock. Sometimes emergent patterns can be scale free, meaning roughly that they are organized similarly at different (e.g., spatial) scales. Examples of scale-freeness include coast lines, naked trees, broccoli, and—as Barabási and Albert claimed—power-law degree distributions. A probability distribution can be scale free in the sense that statements about relative abundance, such as “there are twice as many vertices with degree two thousand than three thousand” are true even if one changes the scale “thousand” to “hundred”, or “million”^5^.

A final concept of complexity science that is important for understanding the success of scale-free networks is universality. Emergent patterns are often consequences of basic symmetries and behavioral rules of the constituents. Thus, bird flocks of different species can have similar movement patterns. This means that rather different systems can share emergent properties—Barabási and Albert argued that the scale-freeness of networks is such a universal phenomenon.

In the Platonic realm of simple mechanistic models, extrapolated to infinite system size, the concepts of emergence, universality and scale-freeness are well-defined and clear. However, in the real world, where systems are finite and many forces affect them, they become blurry. If you meditate in front of a broccoli, you will notice that even though the same principles of organization occur at different scales, there are also differences—you can guess how zoomed-in a picture of a broccoli is. This blurring makes complexity concepts less applicable to the real world, but is that enough to make them uninteresting? I think that is a question without a scientific answer.

Barabási and Albert’s bold view of a simple statistical pattern uniting networks—from power grids to social networks, from neural networks to the Internet—was challenged from early on. Critics made the point that although the degree distribution is scale free, the actual networks are not^5^. They pointed out that power-law degree distributions and the preferential attachment mechanism were already discovered^6^. Even more polarizing, however, was the claim that degree distributions rarely follow power laws.

The first paper that used statistical tests to refute claimed observations of scale-free networks was probably by Jones and Handcock^7^. More influential, however, was the paper by Clauset, Shalizi and Newman^8^ that devised a statistical procedure for evaluating if a probability distribution is a power law or not. Until then, researchers typically plotted distributions in a double-logarithmic scale. If such plots were straight enough, they called it a power law. Replacing that method—inexact and prone to motivational biases—was a great merit of ref. ^8^.

In “Scale-free networks are rare”, now published in *Nature Communications*^9^, Broido and Clauset take a more data-centric approach. They use a collection of 927 empirical networks (compared to seven of ref. ^8^) and exploit the fact that information-rich network data can be reduced to many simple networks. Broido and Clauset proceed to evaluate how close to power laws the degree distributions of different classes of networks are, using five categories of scale-freeness—from “super-weak” to “strongest”. Fifty-seven percent of the data sets, they find, belong to at least some kind of scale-free class, while only 4% belong to the “strongest” category. Furthermore, while biological and technological networks can reach the “strongest” level, social networks can, at most, be “weakly” scale-free.

When “Scale-free networks are rare” appeared as a preprint in January 2018 it triggered a tremendous online activity, including articles, blog posts (by Barabási https://www.barabasilab.com/post/love-is-all-you-need among others), and lengthy discussions on social media. The most common arguments against the claim that scale-free networks are rare relate to the concept’s origins in complexity science and in particular the fact that scale-freeness is only well-defined in the infinite-size limit. According to this view, a network is scale-free if its degree distribution approaches a power law as the network keeps growing following the same mechanisms. Thus, fluctuations that make a network fail a statistical test would not matter, because if one let the network evolve, it could soon pass the test (or vice versa—pass the test for small networks and fail for large)^10^.

Now we have one camp of network scientists thinking of scale-free networks as ideal objects in the large-size limit, and another seeing them as concrete objects belonging to the real world. Scientists often sneer at the humanities with their schools of thought and lack of consensus^11^, but such is the current state of network science.

Can we find consensus? A first step would obviously be to agree on a precise definition. Maybe we could define scale-freeness as an emergent property and find a principled, statistical way of testing for it? We would first have to infer the growth process, then extrapolate it to infinity. Without such scaling arguments, methods (like that of ref. ^9^) are restricted to statements about finite data sets. Inferring general network growth processes is a challenging and relatively unexplored topic^12,13^. Ref. ^14^ probably represents the state of the art. Furthermore, for networks where the growth is not documented, this approach is, strictly speaking, impossible. Instead of striving for a synthesis of the two schools, we could also keep both. Distinguishing between statistical and emergent scale-free networks should be easy since it is equivalent to the distinction between finite, real networks and their projections into infinity.

On one hand, we need the large-system limit, if not for the allure of complexity-science buzzwords, then for the mathematical concepts derived in this limit. These include concepts that have spread far outside the mathematical sciences. One such example is epidemic thresholds—models of epidemics can change behavior (from one state where large-scale outbreaks are possible, to another where all outbreaks will be small) at exact parameter values (governing, e.g., how easily a person gets infected). A celebrated result states that scale-free networks with degree exponents less than three don’t have epidemic thresholds^15^, i.e., epidemics can always reach a large fraction of the population. However, this holds only for infinite networks. If, by the method of Broido and Clauset, a finite network is consistent with a power-law degree distribution of exponent two, then it still, strictly speaking, does not have an epidemic threshold. Applying methods for finite networks to emergent properties will always be inexact, just like bringing emergence-related concepts (such as scale-freeness) to the real world of finite networks.

On the other hand, we need theories and methods that—in the same vein as Broido and Clauset—are specialized to finite networks. The reason is simple—network science is successful because it is applicable to real-world network data, and real-world data may be large, but never infinite.

Why do we still debate scale-free networks? By January 2018, the polemics following ref. ^8^ had long since subsided. Presentations at network-science conferences reporting new scale-free network findings, or models of their emergence, were rarer than the scale-free networks themselves (viz. 4%). I, and (I believe) most colleagues, were following the principle that “knowledge of whether or not a distribution is heavy-tailed is far more important than whether it can be fit using a power law”^13^. Thus, it was surprising that the scale-free debate would flare up again. There are obviously scientific reasons for it. The preprint of ref. ^9^ has, at the time of publication, over 50 citations. One such spin-off work is Voitalov et al.’s^10^ explorations of softer statistical criteria for scale-freeness. Still, it often feels like the topic of scale-free networks transcends science—debating them probably has some dimension of collective soul searching as our field slowly gravitates toward data science, away from complexity science.
